# Examining the Associations of Social Support on Later‐life Cognition in the Health and Aging Brain Study‐Health Disparities (HABS‐HD) Cohort

**DOI:** 10.1002/alz.089092

**Published:** 2025-01-09

**Authors:** Jillian K. Lee, Leigh A. Johnson, Sid E. O'Bryant, Michelle M. Mielke, HABS‐HD Study Team

**Affiliations:** ^1^ Wake Forest University School of Medicine, Winston‐Salem, NC USA; ^2^ University of North Texas Health Science Center, Fort Worth, TX USA

## Abstract

**Background:**

Social support has been associated with a reduced risk of heart disease and stroke, higher cognitive function, less cognitive decline, and resilience to stress. Notably, social support differs by gender and race/ethnicity. However, it is not clear whether these factors modify the relationship between social support and cognition. In a large diverse cohort, we cross‐sectionally examined the association between social support and cognition and whether gender or race/ethnicity modified the associations.

**Method:**

Participants included 2,831 older adults (mean [SD] age, 64.98 [8.53] years; 63% women, 39% Non‐Hispanic White, 39% Mexican American, 22% African American) enrolled in the Health and Aging Brain Study‐Health Disparities (HABS‐HD). Perceived social support was measured using the Interpersonal Support and Evaluation List. Comprehensive cognitive testing including multiple tests across the following domains: memory, language, processing speed, and attention/executive function. Linear mixed‐effect models evaluated the associations between social support and cognitive performance, adjusting for age, education, gender, and race/ethnicity, the Geriatric Depression Scale, and the Penn State Worrisome questionnaire. Interactions between social support and gender or race/ethnicity on cognition were assessed.

**Result:**

Higher social support was significantly associated with most tests across the cognitive domains. With increasing social support, women performed significantly better than man on Digit Span Forward (b = 0.0391, p = 0.0019) and Backward (b = 0.0335, p = 0.0055), Digit Symbol Substitution (b = 0.1259, p = 0.03780), and Trails A (b = ‐0.2569, p = 0.0198). Increasing social support was more strongly associated with cognitive performance on the Digit Symbol Substitution for African Americans compared to non‐Hispanic whites (b = 0.1652, p = 0.0346). Race/ethnicity did not modify the association between social support and any other cognitive test.

**Conclusion:**

In the cross‐sectional study, higher social support was associated with better cognitive performance across all domains. Associations appeared to be stronger for women versus men but generally did not differ by race/ethnicity. Future research is needed to evaluate the longitudinal associations of social support and changes in cognitive trajectories overtime.

